# Diabetes Cured by Skim-milk

**Published:** 1878-01

**Authors:** H. W. Jones

**Affiliations:** Chicago


### DIABETES CURED BY SKIM-MILK.

By Dr. H. W. Jones, Chicago.

On the 24th of May last, a gentleman consulted me, whose case presented the following aspects :

He was 42 years of age, of a bilio-nervous temperament, of temperate habits, though using tobacco freely, and with a history of remarkable health, excepting an occasional sick-headache, and a rare colic from indigestion.

During the previous autumn and winter, he had been subjected to severe mental strain, and was often engaged at his desk ten or twelve hours a day, under the pressure of anxious responsibilities. For the preceding ten days or more, he had noticed a decided increase in the quantity of urine passed, believing it to amount to eight or ten pints daily, while the frequency of micturition was a source of annoyance by day and night.

He was also unusually thirsty, experienced decided lassitude, and though with fair appetite, had difficult digestion, and was losing weight. His mouth was aphthous, and he had balanitis for the first time in his life. A specimen of urine, presented the next day, was of a bright amber color, fruity odor, acid reaction, without deposit, specific gravity 1047°, and with Fehling's test precipitated abundant sugar.

The recent knowledge of Dr. Arthur Scott Donkin's plan of treating diabetes by a strict confinement of the patient to the use of skim-milk, led me to propose this regimen as the most promising known, and Mr. D. entered at once upon it with an intelligent appreciation of the gravity of his disease, and of the rules laid down for his guidance. These were briefly as follows : He was (1) to take *no other food* than skim-milk ; for the first day or two (2) he was to drink but *four or five pints* in all, distributing this amount at intervals of two hours ; after the third day (3) he might add a pint or two of the same preparation *curded with rennet*, but he was not to exceed seven pints, including curd.

The milk was obtained through special sources, and was kept till all cream had risen, when every particle of the latter was carefully removed.

The record here given, Mr. D. kept personally, and it is replete with interest.

| DATE. | SP. GR. | SUGAR. | NO. PINTS OF MIIK 24 HOURS. | NO. PINTS URINE 24 HOURS. | WEIGHT. | REMARKS. |
|---|---|---|---|---|---|---|
| May 24 | 1047° | heavy | .... | 8 to 10 | .... | Disease discovered. |
| May 25 | .... | .... | .... | .... | 154 | Usual weight about 165 pounds. |
| May 26 | 1047° | heavy | 3 | .... | .... | Began skim-milk ; other food and drink given up. |
| May 27 | .... | .... | 6 | 6 | | |
| May 28 | .... | .... | 7 | 5 | 147 | Weak and indisposed to exertion. |
| May 29 | .... | .... | .... | 4 | 150 | |
| May 30 | .... | .... | 8 | 4 | 152 | Two pints made into curd. |
| May 31 | 1038° | less | 8 | .... | 153 | |
| June 1 | .... | .... | 8 | .... | 154 | Constipated. |
| June 2 | .... | .... | 8 | 3½ | 154 | |
| June 7 | 1030° | less | 10 | .... | .... | Four pints made into curd. |
| June 10 | .... | .... | .... | 4 | | |
| June 14 | 1023° | little | .... | .... | 152½ | Water enemas relieve constipation. |
| June 21 | 1022° | little | 10 | .... | 152 | |
| June 28 | 1018° | none | | | | |
| July 1 | .... | .... | .... | 2 | | |
| July 5 | 1032° | sugar | 10 | .... | .... | Milk had not stood long enough to separate cream. |
| July 7 | 1028° | less | 10 | | | |
| July 8 | .... | .... | 8 | 3 | | |
| July 10 | 1022° | none | 8 | 2½ | | |
| July 12 | 1020° | none | 10 | .... | .... | From this date no sugar appeared ; the same food being continued. |
| Aug. 7 | 1018° | none | .... | .... | .... | Began eating mutton-chops and beef; no starchy foods. It is four weeks since sugar disappeared, and seventy-three days since treatment began. Averaged 1020° to 19th. |
| Aug. 19 | 1022° | none | .... | .... | .... | Eats now lamb, mutton, beef, fish, cabbage, onions, squash, oysters, eggs, chicken, and drinking tea and coffee. |
| Aug. 30 | 1020° | none | 3 | normal | 145 | |
| Sept. 10 | 1020° | none | 3 | normal | .... | Commenced eating bread. |
| Sept. 20 | .... | ...slight | .... | .... | .... | A sleepless night and much anxiety. |
| Sept. 21 | .... | none | | | | |

It will be observed that in five days the specific gravity of the urine diminished from 1047° to 1038°, and its quantity, from eight pints or more, to four pints daily. Meantime, the weight of the patient suffered little diminution.

In less than thirty days the specific gravity reached 1018°, and sugar wholly disappeared ; but July 3d, when the atmosphere was unfavorable to the preservation of milk, and to the total removal of cream, sugar reappeared, but in very slight amount, the density meanwhile reaching 1032°. By the 10th, however, this fell again to 1022°, and the quantity of urine passed, to two and a half pints.

Four weeks from this time he began to eat mutton and beef, but touched no amylaceous food.

One month later he was permitted bread, and found no sugar during about a week, but then. after a sleepless night, and much anxiety, he reported "a trace," which disappeared in less than a day, and has never since recurred.

At present, the patient seems in perfect health, though he has been actively engaged at his usual avocation during the entire treatment. His stomach was never more dutiful, and he has had neither sick-headache nor colic since May last.

The result of this case, as compared with previous experiences in the treatment of diabetes, leads the writer to hope that Dr. Donkin's views* may be more widely recognized as both scientific and practical, and that the record of other cases treated in accordance, may prove as satisfactory.

* "Diabetes and Its Relation to Food," and "Diabetes and Its Treatment by Skim-Milk."

